# Change in Australian Vitamin A Intakes over Time

**DOI:** 10.1093/cdn/nzz081

**Published:** 2019-07-12

**Authors:** Angela E Messina, Tracy L Hambridge, Dorothy E M Mackerras

**Affiliations:** 1 Food Standards Australia New Zealand, Canberra, Australia; 2 Department of Science, Medicine and Health, University of Wollongong, Wollongong, New South Wales, Australia

**Keywords:** vitamin A, nutritional requirements, nutrition surveys, within-person variance, diet/statistics and numerical data, National Cancer Institute (US), Iowa, bias, daily-consumed nutrient distribution, Australia

## Abstract

**Background:**

The mean intake of vitamin A of Australians aged 2 y and older was 300 µg retinol equivalents lower in the 2011–2012 national nutrition survey than in 1995 and decreases preponderated in adults rather than young children.

**Objective:**

This aim of this study was to identify the foods associated with this change and to examine how the method used to adjust for within-person variability affects the estimated prevalence of inadequate intakes in both surveys.

**Methods:**

Foods contributing to vitamin A intake were calculated from the first day of data. The prevalence of inadequate intakes was calculated using a 2-d average, the Iowa State University method, and the National Cancer Institute (NCI) method and either taken from the published reports or calculated using Food Standards Australia New Zealand's in-house software.

**Results:**

In adults, lower consumption of liver, yellow fat spreads, milk products, and carrots and similar root vegetables accounted for most of the change in intake. Vitamin A intake data were less right-skewed in 2011–2012 than in 1995. The prevalence of inadequate vitamin A intake depended on the adjustment method chosen: for example, in 2011–2012 it ranged between 3% and 55% in men aged 19–30 y. The NCI method prevalence (21% for this group) is taken as the preferred estimate of inadequacy because the method adjusts around the mean and accounts for several other sources of variance. However, the NCI method could not be used to analyze the 1995 survey.

**Conclusions:**

The lower vitamin A intake in Australia was related to changes in retinol intake rather than carotenoid intake and to lower consumption of several different types of food. The estimated prevalence of inadequate intake depends on the statistical method chosen for analysis. A direct measure of vitamin A status is needed to allow conclusions about the implications of the decreasing intake of this vitamin.

## Introduction

Reference values for vitamin A, a fat-soluble vitamin necessary for normal immune function, vision, gene expression, and reproduction, for Australia and New Zealand were revised and released in 2006 (1). Inadequate vitamin A intake is common in many lower-income countries (2) where deficiency leads to xerophthalmia, night blindness, and an increased risk of morbidity and mortality in young children (3). Mean daily vitamin A intake from food of the Australian population aged 2 y and older was 1123 µg retinol equivalents (RE) in the 1995 National Nutrition Survey (4) and 815 µg RE in the 2011–2012 National Nutrition and Physical Activity Survey (5). Low vitamin A intakes were more common in adults than in young children (5), in contrast to what is observed in many lower-income countries.

We examined 2 issues related to vitamin A intake in the 1995 and 2011–2012 surveys and focused on adults. Firstly, differences in food consumption were examined. We particularly focused on a perception that the consumption of organ meats, which are rich in vitamin A but infrequently consumed, had changed over time. Secondly, we examined the impact of changing statistical methods on the estimated prevalence of inadequate vitamin A intake. Dietary references such as the Estimated Average Requirement (EAR) should only be compared to usual intakes of the nutrient (6). However, large-scale national surveys often collect 1–2 d of intake information from participants and then estimate the distribution of longer-term intake mathematically. A second day of information was available for 10% of the respondents in the 1995 survey and longer-term intakes were estimated using the Iowa State University (ISU) method in reports from the Australian Bureau of Statistics (ABS) (4, 7). A second day of information was available for 64% of the respondents in the 2011–2012 survey and usual intakes were estimated using the method developed more recently by the US National Cancer Institute (NCI) (5, 8, 9). These 2 approaches could have an important impact on assessing trends in inadequate intakes.

## Methods

### Data sources

The 1995 and 2011–2012 national nutrition surveys were similar in that they were a random multistage area sample of private dwellings excluding those located in discrete Aboriginal and Torres Strait Islander Communities and Very Remote Areas. Within the household, sampling covered persons aged 2 y and older (10, 11). The first survey was conducted between February 1995 and March 1996. Dietary information for ≥1 d was collected from 13,858 respondents. The second survey, conducted between May 2011 and June 2012 (except in August and September 2011 when the census was being taken), was a subcomponent of the larger 2011–2013 Australian Health Survey. Dietary information for ≥1 d was collected from 12,153 respondents. A 24-h recall was conducted in the home, using non-English languages when necessary. In the 2011–2012 survey, all respondents were asked to repeat the recall via the Computer Assisted Telephone Interview and more than half (64%) completed this. We divided data for both surveys into age groups to match those used to describe the EARs released in 2006 (1). Appropriate survey sampling weights were used in analyses of the 2011–2012 survey but not for the 1995 survey because the sampling in 1995 was done in age bands which are the focus of the current analysis.

Age or age group; sex; survey sampling weight; intake of vitamin A, preformed retinol, and various carotenoids; and the consumption of all foods by each person for as many days as available were extracted from the 2 data sets (10, 11). The Australian Food, Supplement and Nutrient Database (AUSNUT) food composition tables (12) give data for α- and β-carotene, β-cryptoxanthin, and β-carotene equivalents (BCE)—which is calculated as µg β-carotene + 0.5 (µg α-carotene + β-cryptoxanthin). The unit for vitamin A in Australia is micrograms of retinol equivalents (µg RE), which is calculated as µg retinol + 1/6 µg BCE (1).

### Food consumption patterns

The mean intake of vitamin A, retinol, and BCE provided by various foods and food groups was calculated using the first day of intake data from each survey. We focused on adults (defined as 19 y and older) when examining food groups because vitamin A intake had changed in this age group (**Table 1**). In addition, there were relatively small numbers of adolescent boys in 2011–2012 and this limits the interpretation of results for that age group. AUSNUT is structured into major and minor food groups and individual foods and there is a concordance file to link food codes between the surveys (9). However, it is recognized that the concordance is not perfect for all foods owing to factors such as the changing propensity to code final dishes as opposed to recipe ingredients in the dish.

**TABLE 1:** Mean intake of vitamin A from food from the first day of the survey, consumption of liver, and recalculated mean vitamin A intake after excluding consumption of liver in 2 national Australian surveys, by age and sex^1^

	Number of nutrition survey respondents		Mean intake of vitamin A from the first day of the survey (**µ**g RE)^2^		Mean intake of vitamin A from the first day of the survey, excluding liver^3^ (**µ**g RE)	
Age, y	1995	2011–2012	1995	2011–2012	1995	2011–2012
Males						
2–3	170	165	791	587	791	587
4–8	513	401	798	743	796	743
9–13	474	435	1171	774	1026	774
14–18	378	373	1166	754	1164	754
19–30	1014	1116	1301	866	1271	852
31–50	2080	1757	1271	826	1169	825
51–70	1442	1335	1377	934	1230	841
≥71	545	462	1351	958	1085	868
All males	6616	6045	1243	847	1142	817
Female						
2–3	213	152	675	557	670	557
4–8	464	374	751	575	749	575
9–13	439	426	894	703	894	702
14–18	356	367	1009	625	894	625
19–30	1189	1072	982	749	925	749
31–50	2317	1778	1047	797	947	796
51–70	1577	1379	1145	907	981	817
≥71	687	560	1015	854	933	783
All females	7242	6108	1013	782	923	755
All persons	13,858	12,153	1123	815	1027	786

1 RE, retinol equivalents.

2 The relative SE of mean vitamin A intake ranged from 2% to 6% among the age-sex subgroups in the 2011–2012 survey (5) but could not be modelled for the 1995 survey (4).

3 Liver includes cooked liver, which may include that which was floured before cooking but excludes liver pates and liver dishes (e.g., with vegetables).

### Adequacy of dietary intake

Adequacy of vitamin A intake was assessed by the EAR Cut-point Method, which involves calculating the proportion with intakes less than the relevant EAR for all age-sex subgroups (6). The EAR is a reference value used to assess usual intakes of populations, not 1-d intakes. There are 3 possible ways for deriving an estimate of longer-term intake from the available data. These remove different amounts of within-person variation from the population SD.

The ISU method (7) was available when the 1995 data were released. This involves using both days of data from the subgroup with this information to determine the within- and between-variance components and derive the correction factor which is applied to the total data set for the first day (Day 1). It does not correct for other effects such as day-of-the-week effects. An important constraint with this method is the assumption of a normal intake distribution and previously it was time-consuming and complex to explore multiple possible transformations. The ABS used this method in its publications (4) of the 1995 survey and assumed that other users would also use it. Therefore, the ABS provided the correction factors which assumed that vitamin A intake distributions would be logarithmically transformed (using the natural log) but no other nutrient would be transformed (4, 10). Logarithmic transformation results in adjustment around the median rather than the mean. This method can also be used to calculate adjusted intakes with the 2011–2012 data. However, these data were less right-skewed than the 1995 data and so a logarithmic transformation is not necessarily the right choice. Therefore, the ISU method was calculated with and without logarithmic transformation of the 2011–2012 data, because it is possible that some users might assume that the original ABS advice would apply to subsequent surveys.

The more recent NCI method transforms the data to the best distribution but adjusts around the mean, and includes age, sex, day-of-the-week, and sequence effects (the effect of the data collected in the second 24-h recall compared with day 1; the day 2 data may not be as fully reported) in the usual intake estimate (8). The ABS analyzed the 2011–2012 data using the available SAS (SAS Institute Inc) program (9, 13) but the small number of replicates collected in 1995 does not allow it to be used with those data.

The third method is to average the 2 d of dietary information for the participants who provided this. However, some form of weighting might be necessary because these participants might not reflect the age-sex distribution of the whole sample. This method can be used with the 2011–2012 data because 64% completed the second interview and the ABS has provided weights to use with this subset. However, a 2-d average was not calculated with the 1995 data because only 10% of the total group completed the second recall, leading to small numbers in the age-sex subgroups, and because weights are not available for this subset.

The 2-d average and ISU results described here were generated using Food Standards Australia New Zealand (FSANZ)’s in-house custom software which generates the mean, median, and a range of percentiles for the distribution, as well as allowing percentages below the EAR for the age-sex subgroups to be calculated based on its outputs. The NCI method results for 2011–2012 are taken from the published ABS report (5). Results for the first day alone are also presented to allow the extent of the change in distribution yielded by each adjustment method to be shown.

## Results

In the Australian population aged 2 y and older, mean intake of dietary vitamin A was 1123 µg/d in 1995 and was 308 µg/d lower in 2011–2012. This change occurred in all age-sex groups aged 9 y and older to varying degrees (Table 1). The change in vitamin A intake comprised a change of 255 µg preformed retinol and of 310 µg BCE (i.e., 52 µg RE).

### Contributors to changing vitamin A intake

**FIGURE 1:**
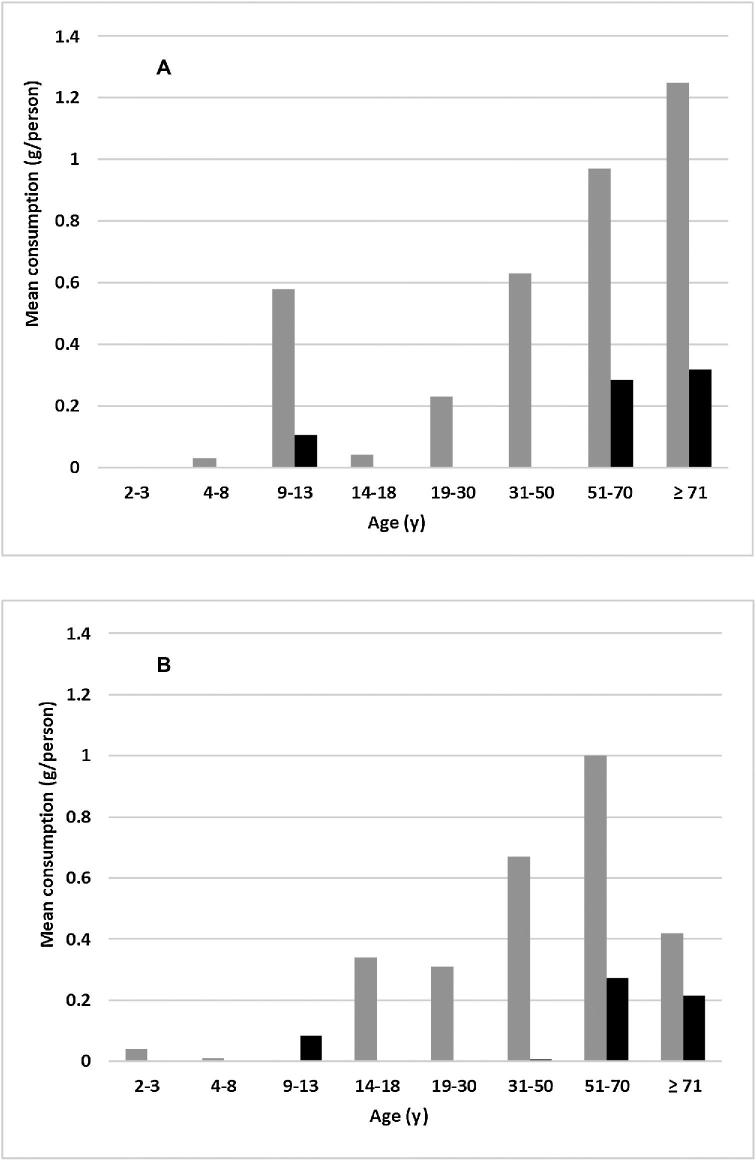
Mean liver consumption on the first day of the survey (grams per person) for all male (A) and female (B) respondents in 2 national Australian surveys. Grey columns: 1995 National Nutrition Survey; black columns: 2011–2012 National Nutrition and Physical Activity Survey.

The proportion of the population aged ≥2 y who consumed liver was 1.1% in 1995 and 0.1% in 2011–2012, although the range of amounts consumed was little changed. Consequently, the mean consumption of liver, when averaged across the entire population, was lower in 2011–2012 (**Figure 1**). Consumption was higher in males than females and was small in those aged <30 y in 1995 and <50 y in 2011–2012, which indicates that younger birth cohorts did not choose this food. Across the entire population, the change in liver consumption accounted for approximately one-fifth of the change in vitamin A intake by adults and one-quarter of the change in preformed retinol intake between the surveys. However, the change in BCE intake was not related to liver consumption because liver contains very little provitamin A and there was no difference in vitamin A intake between the surveys after calculating the equivalent vitamin A from BCE and rounding.

Foods contributing to the vitamin A intake were considered in greater detail in adults aged 19 y and older (**Tables 2** and **3**). In this age group, vitamin A was lower by 326 µg RE/d, retinol by 261 µg/d, and BCE by 377 µg/d (equivalent to 63 µg RE) in the 2011–2012 survey than in the 1995 survey (Table 3).

**TABLE 2:** Proportion of adult respondents (19 y and older, sexes combined) consuming selected foods, median consumption amount reported by consumers, and mean consumption amount for all respondents in 2 Australian national nutrition surveys

	1995 National Nutrition Survey			2011–2012 National Nutrition and Physical Activity Survey		
Food group and subgroup	Percentage consuming	Median among consumers (g)	Mean/person (g)	Percentage consuming	Median among consumers (g)	Mean/person (g)
Meat, poultry, and game products and dishes	81.2	144.0	157.4	69.8	174.0	153.0
Liver	1.3^1^	18	0.68	0.1^1^	104	0.12
Fats and oils	75.3	12	12.2	45.0	9.5	5.6
Butters and dairy blends	23.1	10	3.5	17.9	9.6	2.2
Margarine and table spreads	53.1	10	7.8	25.9	9.5	2.9
Milk products and dishes	93.3	247.7	289.3	84.5	187.2	220.8
Dairy milk (cow, sheep, and goat), unflavored	83.3	187	203.5	69.0	128.8	139.1
Yoghurt	8.6	155	13.8	16.0	124.8	23.5
Cream	7.7	20.5	2.9	3.8	26.0	1.8
Cheese	41.2	24	14.6	31.3	25.0	11.4
Frozen milk products	15.7	82.7	17.6	13.0	83.0	14.6
Dairy substitutes	2.3	191.7	5.0	4.7	185.4	9.7
Milk substitute (all)	2.2	198.8	5.0	—	—	—
Nonalcoholic beverages	100.0	1785.3	1983.4	99.7	1750.0	1938.6
Fruit products and dishes	56.3	200.0	143.5	58.1	190.6	142.3
Vegetable products and dishes	88.8	250.0	258.8	77.2	162.9	171.5
Carrot and similar root vegetables	39	42	21.8	17.8	50.4	14.7
Leaf and stalk vegetables	38.2	29	16.8	20.7	24.6	7.0
Mixes of ≥2 vegetables (includes mixed salad leaves)	8.0	77.0	8.1	26.0	100.0	31.9
Pumpkins	13.9	80.6	13.0	5.1	70.0	4.6

1 There were 141 adults who consumed liver in the 1995 survey and 12 in the 2011–2012 survey.

**TABLE 3:** Vitamin A intake from selected foods by all adult (19 y and older, sexes combined) respondents in 2 Australian national nutrition surveys^1^

	1995 National Nutrition Survey			2011–2012 National Nutrition and Physical Activity Survey		
Food group and subgroup	Vitamin A (**µ**g RE)	Retinol (**µ**g)	BCE (**µ**g)	Vitamin A (**µ**g RE)	Retinol (**µ**g)	BCE (**µ**g)
Total, all foods and beverages	1178	579	3587	852	318	3210
Meat, poultry, and game products and dishes	180	166	87	99	55	264
Liver	87	86	1	37	37	0.04
Fats and oils	116	105	68	43	40	19
Butters and dairy blends	38	34	23	19	18	5
Margarine and table spreads	73	66	42	23	21	14
Milk products and dishes	162	148	88	116	104	73
Dairy milk (cow, sheep, and goat), unflavored	64	59	32	44	42	16
Yoghurt	3	2	2	7	6	5
Cream	15	13	8	5	5	3
Cheese	45	40	25	24	21	13
Frozen milk products	20	18	13	23	19	24
Dairy substitutes	1	1	0	3	3	0
Milk substitute (all)	1	1	0	3	3	0
Nonalcoholic beverages	27	3	143	42	21	131
Fruit products and dishes	39	1	226	45	0.3	266
Vegetable products and dishes	447	29	2507	304	8	1779
Carrot and similar root vegetables	273	1	1637	164	0.04	984
Leaf and stalk vegetables	11	0	64	13	0	75
Mixes of ≥2 vegetables (includes mixed salad leaves)	12	0.1	72	75	2	440
Pumpkins	64	1	377	5	0	32

1 BCE, β-carotene equivalents; RE, retinol equivalents.

Although the median amount of liver consumed by adult consumers was much higher in the 2011–2012 survey, which is likely to be due to the small number of consumers (*n* = 12), the range of amounts was the same in both surveys and the mean consumption across all adults was little changed over time (<1 g/person) (Table 2). However, the change in liver accounted for a little more than half the change in retinol from the broad meat products and dishes group. The higher BCE intake in the broad meat group in the 2011–2012 survey is due to higher intake of dishes containing meat and vegetables which are coded into the broad meat group.

The mean consumption of butter and spreads and dairy products changed between the surveys for different reasons (Table 2). The proportion consuming butter and spreads was 30–50% lower in 2011–2012 although the median amount consumed per day by consumers did not change appreciably (Table 2). Note that this does not include oils and fats from mixed dishes. By contrast, the change in the proportion consuming milk was smaller but the median amount consumed by consumers was also lower by about one-third. Although the proportion who consumed milk analogues doubled, this did not offset the lowered proportion consuming milk. The lower intake of 73 µg RE from fats and oils and 46 µg RE from milk products and dishes accounted for 36.5% of the difference in vitamin A intake between the surveys.

The proportion consuming several types of high-carotenoid vegetables (namely the carrot and similar root vegetables, including sweet potato) in 2011–2012 was half that seen in 1995 (Table 2), with a consequent difference in vitamin A intake. However, this does not include all vegetables eaten because the rows in Table 2 describing vegetables capture only vegetables reported as eaten alone. In particular, there was a higher intake in BCE from mixtures of vegetables between the surveys such as salads, mixed frozen or canned vegetables, mixed fresh vegetables that are cooked, and Asian green stir fries in the 2011–2012 survey. As aforementioned, vegetables consumed in a mixed dish with meat are captured under the broad meat heading and also in some cereal-based mixed dishes or in soups.

There was a small increase in vitamin A intake from fruit consumption. The small increases in vitamin intake from retinol from nonalcoholic beverages were related to consumption of chocolate-flavored fortified powders which are typically mixed into milk.

### The prevalence of inadequate dietary vitamin A intake

**FIGURE 2:**
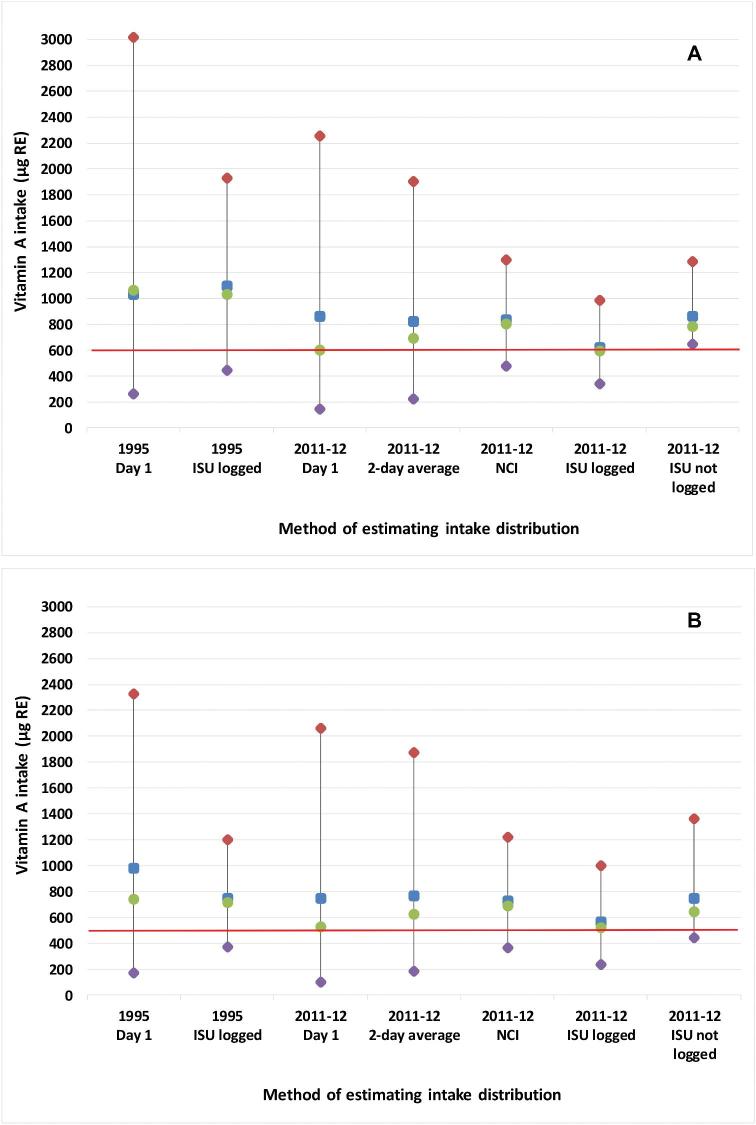
Distribution of vitamin A intake from food on the first day, and after estimation of longer-term intakes by several methods and comparison to the EAR in 2 Australian surveys in men (A) and women (B) aged 19–30 y. Red diamond: 95th percentile; blue square: mean; green dot: median; purple diamond: 5th percentile; the red line indicates the EAR for these groups. EAR, Estimated Average Requirement; ISU, Iowa State University; NCI, US National Cancer Institute.

The lower mean intake of vitamin A in the 2011–2012 survey (Table 1) resulted in a higher proportion of the population with inadequate intakes. However, it is not straightforward to quantify the prevalence or changes in the prevalence owing to the number of ways of correcting for within-person variability. **Table 4** shows how the estimated prevalence of inadequate intakes depends on the statistical method chosen to adjust for within-person variability. **Figure 2** shows how different adjustment methods affected the distribution of vitamin A intakes for young adults aged 19–30 y. This pattern was typical of that seen in the other age-sex groups as well.

**TABLE 4:** Estimated proportion with inadequate dietary vitamin A intakes in 2 Australian national surveys using various adjustment methods^1^

		Survey and adjustment method						
		1995 National Nutrition Survey		2011–2012 National Nutrition and Physical Activity Survey				
Age, y	EAR^2^ (**µ**g RE)	Day 1 only	ISU method,^3^ log adjusted	Day 1 only	2-d average	ISU method,^3^ log adjusted	ISU method, untransformed	NCI method^4^
Males								
2–3	210	6	0	19	6	4	0	1
4–8	275	8	3	19	12	5	0	2
9–13	445	20	10	39	28	26	20	5
14–18^5^	630	30	21	57	55	71	45	33
19–30	625	25	14	51	44	56	3	21
31–50	625	28	16	50	46	55	17	19
51–70	625	28	15	51	42	55	0	17
≥71	625	29	17	48	44	47	0	13
Females								
2–3	210	8	1	14	6	5	0	2
4–8	275	12	2	29	24	10	0	6
9–13	420	21	9	37	30	25	0	9
14–18	485	31	15	48	55	49	32	27
19–30	500	32	17	46	38	44	15	20
31–50	500	29	14	47	36	38	0.3	15
51–70	500	27	10	43	33	36	0	11
≥71	500	26	10	47	38	44	0	15

1 Estimated as percentage below the EAR. ABS, Australian Bureau of Statistics; EAR, Estimated Average Requirement; ISU, Iowa State University; NCI, US National Cancer Institute; RE, retinol equivalents.

2 Released in 2006 (1).

3 Intake distribution was log transformed before making the correction, then exponentiated.

4 Data taken from (5).

5 The ABS notes a higher margin of error >10 percentage points for this age group in the 2011–2012 survey, which should be considered when using this information (5).

## Discussion

Overt vitamin A deficiency is rare in developed countries including Australia, usually occurring in isolated areas as opposed to the general healthy population. Our analysis found that intake was lower to varying extents across the population aged 9 y and older. This was mainly due to lower intake of preformed retinol and related to changing consumption of several different types of foods—margarine and spreads, various dairy products, and liver. The contribution from vegetables, but not fruit, also changed. In the United Kingdom, the proportion of people who consumed liver in their 2000/2001 national survey was half that reported in their 1986/1987 survey (14), which is consistent with our finding about the change in consumption of this food.

Underreporting of food consumption could be an explanation of this finding (15). Based on the expected energy intakes in relation to measured body weights, underreporting seems to have been greater in the 2011–2012 survey than in the 1995 survey. Women were more likely to be under-reporters in 1995 than men, but in 2011–2012, the proportion of male under-reporters was higher (9). It is possible that energy intake is underreported by 17–20% in the 2011–2012 survey (9). Social desirability bias suggests that foods which are promoted by health educators as “less healthy” are more likely to be underreported relative to “healthy” foods. Because “unhealthy” foods do not tend to be good sources of vitamin A, underreporting may be less influential for this vitamin than for some other food components. Industry data indicate an ongoing decline in yellow spread use (16, 17). The range of low-fat cheeses available to Australian consumers increased between the surveys. There has been some regrouping of foods between surveys (9), and differences in how many foods were recorded as individual recipe ingredients rather than fully prepared foods. Although this would affect the apparent contribution of the various food groups, it would not affect the overall mean intake of the vitamin. Therefore, it seems reasonable to conclude that vitamin A intake was lower in Australia in 2011–2012 than in 1995, even if the extent is difficult to quantify.

Voluntary fortification of margarine and edible oil spreads with vitamin A is permitted in Australia and the brand named products typically take up this permission and are fortified. Although voluntary fortification of certain milks, cheese, and selected other foods with vitamin A is also permitted (18) in Australia, these permissions are little used. An exception are the plant-based milk analogues, which are generally fortified with a range of nutrients in Australia. However, these products are not widely consumed (Table 2).

We have not included vitamin A derived from supplements in our analysis. Firstly, detailed information about usage was not collected in the 1995 survey. Although this information was collected in the 2011–2012 survey, adding it to the dietary intake alters the shape of the distribution such that it violates underlying assumptions of the NCI method model (on the distribution of variances) and yields nonsensical results (9). However, in the 2011–2012 survey, users of any type of supplement tended to have higher intake of a range of micronutrients than those who did not use supplements (19). Hence, including supplemental vitamin A in the analyses might not have much impact on the proportion of the population with low intakes in 2011–2012.

We estimated the proportion with inadequate vitamin A intakes by a variety of statistical methods to correct for within-person variability. All methods of adjustment reduced the spread of the distribution compared with the Day 1 data and this affected the estimated prevalence of inadequate intakes (Figure 2). The 2-d average was the least effective. Using a logarithmic transformation in the ISU method changes the resulting mean because it adjusts around the median and this shifts the distribution downwards compared with methods which adjust around the original mean. Differences between the NCI and ISU method results are also related to exactly which transformation is used, because different transformations could be chosen for different age groups in the NCI calculations, whereas we stipulated the same transformation for all groups with the ISU method. In addition, the NCI method corrects for several additional factors compared with the ISU method. Therefore, it is not possible to make unqualified statements about how these 2 methods compare. The ABS and FSANZ have chosen to use the NCI method when estimating the prevalence of inadequate intake in the 2011–2012 survey because it retains the mean, adjusts for additional factors, and simplifies choosing the normalizing transformation for each subgroup compared with the ISU method. Both agencies use the same seed for generating the Monte Carlo routines that are used in the NCI method. The automation of testing and selecting the most appropriate transformation for each modelled group (e.g., children to 8 y, males ≥9 y, females ≥9 y) also makes the method more convenient than having to check various possible transformations with multiple population groups. However, a disadvantage of this method is that the only publicly available macro is in the SAS programming language (13).

Our results highlight a conundrum when trying to monitor trends in population nutrient intakes. It is desirable to adopt advances in survey methods for collecting and analyzing data but these methods cannot always be retrofitted to previously conducted surveys. In our case, the lack of sufficient replicates in the 1995 survey means the NCI method cannot be used. Consequently, the most appropriate analysis for each survey has to be chosen, as was the case for the comparisons presented in this article, even though this introduces a degree of uncertainty around the comparisons. This highlights the need to examine the methods used by others when comparing different reports and to avoid over-interpreting apparent changes without looking carefully at the impact of methodological and other differences.

The origin of the EAR is another consideration when interpreting the implications of the low intakes. As is the case with many nutrients, data are sparse and there is extrapolation between age groups. Data from 17 Bangladeshi surgical patients with adequate serum retinol concentrations (20) were a primary influence in increasing the US EAR in adult men by >100 µg/d in 2001 (3), owing to a decrease in the estimated storage efficiency compared with the data which had been used previously. The result was extrapolated to other age-sex groups, and this was subsequently adopted in Australia (1). Although this was the most recent information that could be used, it illustrates the unstable basis of the recommendation. Consequently, there is uncertainty in how much importance to place on the 10–30% prevalence of inadequate intakes in people aged 14 y and older in the 2011–2012 survey.

It is worth noting that the EARs used in Australia are numerically the same as those used in the United States but the units are different (1, 3). Compared with the RE calculation used in Australia and this article, the American retinol activity equivalent halves the contribution of BCE to vitamin A intake. This would have decreased the mean intake of vitamin A in our surveys and increased the prevalence of inadequate intakes. In addition, it would have down-weighted the contribution of plant foods to vitamin A intake at each point in time and to the difference over time. The bioavailability of the provitamin A carotenoids is another area of uncertainty in the calculation of vitamin A intakes. Although it is clear that the bioavailability of vitamin A varies across foods, it can be debated whether a single conversion factor should be used for all foods or whether multiple conversion factors, depending on the food matrix and other dietary factors, would be more appropriate.

Although it seems clear that intake of vitamin A was lower in 2011–2012 than in 1995, it is difficult to quantify the current prevalence of inadequate status because neither survey included a direct measure of vitamin A status. Our analysis justifies including this vitamin in the next biomedical survey. However, measurement needs to go beyond serum retinol, which is homeostatically controlled until liver stores are very low (21). Surveys of selected population subgroups over the last few decades in Australia have found adequate serum retinol concentrations (22–25) even when carotenoid concentrations are low (26). Consequently, an alternative, or additional, valid measure of liver reserves (21) would be more informative in the Australian population.

In conclusion, the mean intake of vitamin A in Australia was lower in 2011–2012 than in 1995 and it seems unlikely that this could be solely attributed to a greater degree of underreporting in the 2011–2012 survey or to a change in the statistical analysis method given the change in consumption of relevant foods. A change in the intake of preformed retinol, not provitamin carotenoids, accounted for the majority of the change in vitamin A intake. The prevalence of inadequate intakes was higher in adolescents and adults than in younger children and ranged from 11% to 33% when estimated using the NCI method to correct for within-person variability. An exact quantitative assessment of the trends in the prevalence of inadequate intake is difficult owing to improvements in survey and statistical estimation methods over time. Biochemical data are needed to determine whether the observed intakes reflect true inadequacy in the Australian population or are due to a combination of other factors such as underreporting of food consumption, imprecise bioavailability calculation factors, and sparse data for estimating the EAR.

## Notes

The authors reported no funding received for this study.

Author disclosures: AEM, TLH, and DEMM, no conflicts of interest. TLH and DEMM are employed by Food Standards Australia New Zealand.

The confidentialized record files of the survey data analyzed can be obtained from the Australian Bureau of Statistics (www.abs.gov.au).

Abbreviations used: ABS, Australian Bureau of Statistics; AUSNUT, Australian Food, Supplement and Nutrient Database; BCE, β-carotene equivalents; EAR, Estimated Average Requirement; FSANZ, Food Standards Australia New Zealand; ISU, Iowa State University; NCI, US National Cancer Institute; RE, retinol equivalents.
